# Welfare of Workers

**Published:** 1920-04-17

**Authors:** 


**THE PUBLIC WELL-BEING—(*continued*).**

### Welfare of Workers.

### Scientific Research and Assistance.

An interesting scheme is on foot for the founding of a National Institute of Psychology and Physiology as applied to industry. Dr. George H. Miles is acting as secretary, and the proposal is supported by great leaders of industry, as well as by psychologists and physiologists like Dr. C. S. Myers, Professor C. S. Sherrington, and Prof. E. H. Starling, and by educationists like Sir Robert Blair.

One of the chief proposals is the establishment of a well-equipped laboratory or laboratories for research into various occupations, in order to determine the conditions necessary to get maximum output with minimum of fatigue and discomfort to the worker, such as elimination of unnecessary movements; and to study the causes of reducing it. Tests would be made in the laboratory for the purpose of establishing standards by which workers can be selected for the occupations for which they are best fitted, mentally and physically. These standards would enable parents and after-care committees to be advised as to the best vocations for children, and would thus eliminate much waste at the outset, and prevent the discontent which arises when a worker finds out too late that he has taken up an unsuitable occupation and is a square peg in a round hole. The facts established by research would be collected and classified, and from time to time published in such a form as to bring out their practical value.

This would be a valuable corollary to the work of the Board of Education, which has called for a continuous record of the health of school children right on through the secondary schools. These children will leave school with an accurate and detailed health record, and if the proposed institute were established it would be enabled to set side by side with these health conditions the scientific results of investigation as to what are the physical and health requirements for entry into particular occupations.

Another function of the institute would be to provide training courses and lectures for investigators, managers, foremen, and welfare-workers in the practical applications of psychology and physiology, and it would undertake investigations at factories and offices in relation to any special problem. It would study the conditions that tend to promote the health, comfort, and welfare of the worker, and also the psychological relations between employer, manager, foreman, and worker, with special reference to securing harmony and co-operation. It would further undertake propaganda work among employers and employed, and co-operate actively with organisations of both, for the furtherance of national unity and prosperity.

The minimum income thought necessary is £6,000 a year for the first five years, but the more money it has the larger can be the initial scale of work undertaken by the institute. A laboratory will be established in London, and later in such of the provincial towns as may be desirable, and it is intended to secure the services of a number of experts in psychology and physiology who will give their whole time to the work. After five years, it is considered that the institute should, to a large extent, be able to support itself out of the fees which it would charge for its services, as in making mental and physical tests and giving advice as to the suitability of individuals for particular occupations; but it would not work for a profit, and all its income would be applied in promoting its objects.

American Examples.

Altogether, this is an attractive and valuable idea, and in keeping with the many other progressive steps, such as the incorporation of the new Medical Research Council. It is also in keeping with the movement which has made some advance in recent years for welfare work in industrial establishments, but this country seems to be lagging behind the United States in this respect. A short time ago the Home Office issued a report on labour administration in America, and it was there stated that welfare conditions are receiving an increasing amount of attention in the United States, and some of those which may now be imposed in Great Britain by order of the Secretary of State are enforceable by law in certain States of the Union. This is the case in New York and Massachusetts as regards the supply of drinking-water, washing accommodation, dressing-rooms, and lockers, first-aid ambulance rooms, and protective clothing.

Describing the welfare arrangements at a Massachusetts boot factory the report states that recreation and rest rooms, a billiard-room, and barber's shop, and library are provided. The principal feature, however, is a "medical room" on each floor, with nurse's room and hospital room with two or three beds adjoining. There is an "oculist's room" and a "dentist's parlour," the firm's oculist and dentist attending daily for half a day, and the

**THE PUBLIC WELL-BEING—(*continued*).**

consultations, like those of the works doctor, are free. Spectacles and tooth fillings are provided at cost price. "The good results of this system in the improved health, timekeeping, and efficiency are said to be very marked, and the absences on account of sickness have sunk to a strikingly low level. In addition to the visiting doctor there is a full-time medical woman as head welfare supervisor."

Of another factory the report states: "The girls' rest room is like a luxurious lounge, and adjoining is an admirable and well-equipped surgery, which includes several beds for use in case of sickness. The whole is in charge of nurses, and doctors are available at short notice. A beautiful dining-room, fitted with clean white-topped tables and good chairs, is provided for the girls, and meals are supplied to them free of cost. There is also a good dining-room for the men, who pay 10 cents for their dinner. Finally there is a magnificent ball and recreation room."

